# Maintaining operability at a high personal cost – a mixed method study on maternal healthcare workers’ experiences during the COVID-19 pandemic

**DOI:** 10.1186/s12913-025-12337-0

**Published:** 2025-01-29

**Authors:** Magnus Akerstrom, Anna Wessberg, Emina Hadžibajramović, Sofie Graner, Ylva Carlsson, Ola Andersson, Maria Jonsson, Elin Naurin, Malin Veje, Verena Sengpiel, Karolina Linden

**Affiliations:** 1 Region Västra Götaland, Institute of Stress Medicine, Carl Skottbergs Gata 22B, 413 19 Gothenburg, Sweden; 2 School of Public Health and Community Medicine, Institute of Medicine, Sahlgrenska Academy, University of Gothenburg, Gothenburg, Sweden; 3 Institute of Health and Care Sciences, Sahlgrenska Academy, University of Gothenburg, Gothenburg, Sweden; 4 Department of Medicine, Center for Pharmacoepidemiology, Karolinska Institute, Stockholm, Sweden; 5 BB Stockholm, Danderyd Hospital, Stockholm, Sweden; 6 Department of Obstetrics and Gynecology, Region Västra Götaland, Sahlgrenska University Hospital, Gothenburg, Sweden; 7 Centre of Perinatal Medicine and Health, Institute of Clinical Sciences, Sahlgrenska Academy, University of Gothenburg, Gothenburg, Sweden; 8 Department of Clinical Sciences Lund, Paediatrics, Lund University, Lund, Sweden; 9 Department of Neonatology, Skåne University Hospital, Malmö, Sweden; 10 Department of Women’s and Children’s Health, Uppsala University, Uppsala, Sweden; 11 Department of Political Science, University of Gothenburg, Gothenburg, Sweden; 12 Department of Infectious Diseases, Region Västra Götaland, Sahlgrenska University Hospital, Gothenburg, Sweden; 13 Department of Infectious Diseases, Institute of Biomedicine, Sahlgrenska Academy, University of Gothenburg, Gothenburg, Sweden

**Keywords:** Maternal healthcare workers, COVID-19, Work environment, Crisis management, Job demand resource model (JDR), Pandemic

## Abstract

**Introduction:**

The COVID-19 pandemic forced leaders and employees in health care services to take difficult decisions to manage risks associated with employee health and the organizations’ functioning. This study aims to identify the changes in employee working routines, job demands, and job resources within Swedish maternal healthcare during the COVID-19 pandemic, and how these changes affected workload and health.

**Methods:**

Data were derived from the longitudinal COPE Staff study involving midwives and physicians within maternal healthcare. Three focus group discussions with midwives and physicians (*n* = 13), and open-ended survey responses (*n* = 604) during the third wave of the pandemic (January to May 2021) were analyzed using deductive content analysis based on the Job Demands-Resources model. Quantitative measures of workload and burnout from three survey waves, both during the pandemic (January to May 2021 [*n* = 782] and January to March 2022 [*n* = 503]) and after the pandemic (February to March 2023 [*n* = 759]), were analyzed.

**Results:**

Multiple changes in working routines were implemented to adhere to national and local guidelines aiming to decrease the spread of Sars-CoV-2. As a result, midwives and physicians experienced increased job demands, including an increased workload and higher emotional and cognitive demands. To balance these demands, new working routines were introduced, and managers increased their efforts to communicate and support the employees. Collegial support also grew. When surveyed, however, most of the maternal healthcare workers said they experienced a high workload. It was found that between 3–7% likely experienced burn out, while another 10% were at risk of burnout during and after the pandemic.

**Conclusions:**

The pandemic had a large effect on maternal healthcare employees. Strategies and adaptations on an organizational, managerial, and individual level played an important role in modifying the impact on the organization’s operations and employees.

**Supplementary Information:**

The online version contains supplementary material available at 10.1186/s12913-025-12337-0.

## Introduction

During a crisis, organizations need to manage risks associated with both employee health and organizational functioning, often with limited information to hand [1]. The COVID-19 pandemic forced leaders and individuals to take drastic and often difficult decisions on multiple levels. Political leaders had to take measures on a national level, managers on an organizational level in the workplace, families had to take measures at home, and individuals had to take personal measures [2].

During a crisis, an organization must strive to sustain or resume operations while minimizing losses for both internal and external stakeholders. Subsequently, it is wise to ensure that a learning process takes place, facilitating the transfer of valuable insights to future crisis [3]. Leaders within the organizations play a crucial role in the process of resource allocation and coordination of these tasks, including sense-making, decision-making, and learning [4]. The COVID-19 pandemic was no exception, with healthcare managers having to design and implement measures to reduce the effect of the pandemic on healthcare workers [5–7].

In healthcare, the overall impact of the pandemic on perceived working conditions and possibility of recovery differed among medical departments [8]. Maternal healthcare (MHC) cannot be postponed, and life-threatening emergencies may occur suddenly and unexpectedly. Thus, MHC needs to maintain a functioning service at all times [9–12]. However, many changes in working routines were needed for MHC to stick to normal routines as closely as possible while still adhering to national and regional guidelines for reducing the spread of the Sars-CoV2 and providing safe environment for both patients and employees.

In Sweden, these changes included physically separating Sars-CoV2 infected pregnant women from other women, as well as introducing triaging and screening procedures to identify infected cases, restricting visits and stays of partners or next of kin in the wards, implementing routines for personal protection equipment use, and when possible, withholding or delaying care and changing to digital out-patient care meetings [13]. Comparable organizational changes were also made in other countries [10, 14–18].

The effect of the pandemic on employee health and the organization’s functioning depended on the specific measures taken and implemented by the managers and the organization during the crisis. Thus, changed working routines during the pandemic had the potential to affect MHC to a large extent [17] and continue to affect it for a long time thereafter [12]. Many studies show the changed work routines have been associated with increased levels of stress and worry within MHC [9, 14, 15, 19–21]. In Sweden, the lack of personal protective equipment, concern about becoming infected, uncertainty about whether the implemented changes were enough, and challenges in communicating updated routines had a negative effect on MHC employees’ working conditions [13]. A higher perceived workload during the pandemic has also been significantly associated with lower job satisfaction levels and higher levels of work-life conflicts, stress, and burnout [22]. The pandemic reduced the feasibility of patient-centred care, leading to stress and anxiety among the MHC workers [14] and also had a negative impact on obstetrics-gynaecology residents’ training [19, 23, 24]. Along with these reported negative effects of the pandemic, positive effects have also been seen. In Sweden, an increased team spirit and feeling valued by peers were shown to have a positive effect on the MHC employees’ working conditions [13]. Thus, there has been an accumulation of knowledge about the effect of the pandemic on working routines and employee health. However, few studies have investigated the mechanism between changes in working routines and employee health. Such knowledge is needed to ensure learning that may be transferred to future crises [9, 15, 22]. Changes in job characteristics alone cannot fully explain employee health and motivation during a crisis. Instead, the interplay between the demands and resources of the individual, the job, the family, and the organization has been found to predict employee health and motivation during the COVID-19 pandemic [25]. Moreover, regulatory strategies of the individual, the family, the leader, and the organization play an important role in modifying the impact of the pandemic on the employees’ health and well-being [25]. Thus, as well as exploring changes in job characteristics and/or employee health during the COVID-19 pandemic, investigating the interplay between demands and resources and successful regulatory strategies on multiple levels could provide healthcare organizations with knowledge of how to optimize conditions for promoting well-being during a crisis.

This study thus aims to identify potential changes within Swedish MHC workers’ working routines, job demands, and job resources during the COVID-19 pandemic, and how they perceived their workload and health. This will be done using a mixed method approach utilizing focus-group discussions with midwives and physicians and quantitative survey data from the COPE Staff Study.

## Methods

### Study design and population

The current study was performed within the COPE Staff Study, a national longitudinal open cohort study investigating work environment factors associated with short-term, intermediate, and long-term effects on well-being in maternal and neonatal health care workers (administrative and medical staff) [22]. In this study, only data from midwives and physicians working in MHC were used.

A mixed method approach with a convergent design [26] was used to collect and analyze the data and included focus group discussions during the pandemic (fall 2021) and survey responses, including free text answers, of the MHC midwives and physicians during and after the pandemic.

### Focus group discussion

Three focus group discussions were conducted during the fall of 2021, with three to six participants in each discussion. The participants worked in labour wards, postnatal wards, and in specialist antenatal care at four different hospitals spread throughout Sweden (two tertiary hospitals and two local hospitals). To ensure variation, the participants had a working experience of between 2.5 and 30 years within MHC. Midwives (*n* = 9) were recruited by open invitation at their workplace and physicians (*n* = 4) were recruited by a direct invitation. Five physicians were invited, of whom one declined participation. All the participants received written and oral information about their right to withdraw consent at any time without stating a reason and signed an informed consent form before participating in the focus group discussion. The focus group discussions were conducted by a team of one trained research assistant and one researcher, and lasted 74, 75, and 85 min respectively. All discussions were conducted by video meeting and the sound was audio recorded and transcribed verbatim. The interview guide (supplementary file 1) was developed specifically for this study and discussion started with the open question ‘How has the COVID-19 pandemic affected your work situation?’ and continued with follow up questions according to the responses.

### Quantitative survey

Quantitative measures of workload and burnout among MHC midwives and physicians were collected from the first three measurements in the COPE Staff Study: January to May 2021 (*n* = 782, 51% midwives) and January to March 2022 (*n* = 503, 54% midwives) during the COVID-19 pandemic, and February to March 2023 (*n* = 759, 60% midwives) after the pandemic; see Table 1 [22].

**Table 1:** Demographics for the three survey waves

Survey	1	2	3
Data collection, year (months)	2021 (Jan-May)	2022 (Jan-March)	2023 (Feb-March)
Pandemic status	Pandemic	Pandemic	Post-pandemic
Total number of respondents, n (%)	782	503	759
Gender, n (%)			
Female	716 (92)	465 (93)	710 (94)
Male	59 (7.5)	35 (7)	45 (6)
Other	4 (0.5)	0 (0)	2 (0.3)
Age, mean (range)	45.1 (26–73)	46.7 (28–78)	46.7 (29–79)
Profession, n (%)			
Midwives	402 (51)	274 (54)	455 (60)
Physicians	381 (49)	231 (46)	305 (40)

For these analyses, survey items from previously published surveys concerning the respondent’s overall workload and level of burnout were used. A single-item question was asked about the respondent’s overall workload, “How would you rate your overall workload?”, with response options on a five-point Likert-scale of very high, high, neither high nor low, somewhat low, and low [22]. In addition, the level of burnout was measured using the 23-item BAT questionnaire [27]. Four MHC employees had not responded to the items concerning the respondent’s overall workload and 83 had not answered one or more of the 23 BAT items, resulting in missing data for the total BAT score. Consequently, 33, 22, 19 and 17 employees were excluded for the four subscales of Exhaustion, Cognitive impairment, Mental distance, and Emotional impairment, respectively.

### Analytical strategy

Data analysis was performed in a stepwise procedure. The first step involved an analysis of the focus group discussions and open-ended survey responses (*n* = 604) during the third wave of the pandemic (January to May 2021). The written responses to three questions from the baseline survey were analyzed: 1. What important changes in the way you work have you implemented that are worth keeping? 2. Is there anything you would like to share with us regarding your work environment during the COVID-19 pandemic? 3. What has had the greatest impact on your work environment during the COVID-19 pandemic?

The interviews and open-ended survey responses were analyzed by deductive content analysis according to Elo & Kyngäs [28]. The Job Demands-Resources (JD-R) model [25] guided the analysis, in which data were coded into job demands or resources respectively, and then grouped and categorized. An example of the qualitative analysis process is shown in Table 2.

**Table 2:** Examples of the qualitative analysis process

Theme	Subtheme	Category	Code	Quote
Maintaining operability at a high personal cost	Job demands	Amount of work	Workload	*“So, it usually requires being with the patients in a completely different way. They need more… they need to talk much more, you notice”*

In a second step, descriptive analyses were carried out by calculating the percentage of survey respondents for each study wave reporting a very high or high workload and by calculating the percentage of respondents at risk of burnout and likely burned out, for both the BAT-23 score and for the four subscales of Exhaustion, Cognitive impairment, Mental distance, and Emotional impairment. The percentage of respondents at risk of burnout and likely burned out for the total BAT-23 score and the four subscales was based on pooled clinical cut-off values (2.59, 3.06, 2.10, 2.70, and 2.30 for at risk of burnout and 3.02, 3.31, 3.30, 3.10, and 2.90 for likely burned out respectively) as suggested by Schaufeli et al. [29]. Differences between midwives and physicians were tested separately using Chi2-tests within each survey.

## Results

### Changes in working routines, job demands and job resources during the COVID-19 pandemic

One overarching theme, *Maintaining operability at a high personal cost*, was identified in the deductive qualitative analysis, with seven categories distributed within the two subthemes: *Job demands* and *Job resources* (Table 3). The overarching theme captures the personal cost to midwives and physicians in maintaining operability within the MHC in an extreme situation. The subthemes and categories are described below.

**Table 3:** Result of the qualitative analysis

Theme	Subtheme	Category
Maintaining operability at a high personal cost	Job demands	Amount of work
		Emotional demands
		Cognitive demands
	Job resources	Working methods and tools
		Competence and staffing
		Possibility of control
		Peer support

#### Job demands

The subtheme *Job demands* consists of identified job aspects that required sustained physical and/or psychological effort and are associated with physiological and/or psychological costs. The subtheme includes three categories: *Amount of work*, *Emotional demands,* and *Cognitive demands*.

##### Amount of work

When restrictions were introduced in Sweden the healthcare system needed to change working methods, as well as introduce additional tasks to minimize the spread of the virus and adhere to the national guidelines. These changes resulted in a perceived increased workload for the MHC workers.

Additional work tasks were introduced, with a requirement to test the women on the ward, their partners, and employees for Sars-CoV-2. Initially, it was unclear how, when, and who to test.

“*Both because there are more things to do, more checks, more samplings, more….” -Midwife in focus group interview 2*

Another additional work task arose from the national directives for maintaining distance and limiting the number of individuals in confined spaces. For the MHC, this meant that partners were not allowed to accompany them through all parts of the MHC chain. These restrictions were perceived negatively by the women and their partners, and the employees found it difficult to inform them in this regard. Having to deal with sad and angry women and their partners affected the employees, both in terms of additional work tasks and the emotional demands this imposed, as illustrated below.

*“So exactly. And then that partners haven’t been allowed to accompany them down to the post-natal ward. This has really been a huge issue for the partners. And many… so many times we’ve had to… well, explain over and over again why we have it like this.” – Midwife in focus group interview 2*

The absence of partners in the post-natal ward was not experienced as all negative, in fact the employees describe having more personal meetings with the women, and that the wards became calmer without women or partners walking in the corridors. At the same time, the employees experienced an increased amount of work in providing the women with the support their partners had normally been giving them.

*“Actually, it often requires being with the patients in a completely different way. They need more… they need to talk much more, you notice. And just these little things, like when they… well, maybe especially those who have had a cesarean section, they have a tough first day where they can barely get out of bed themselves. You need to have, like… the frustration when they lie there and can barely get out of bed, and the baby is lying next to the bed screaming and needs, you know… instead of a partner who could have been there and done it. So, it’s meant that we’ve had more tasks in a different way because we have had to be their… well, help, you know.” – Midwife in focus group interview 3*

The need to communicate and receive information on updated routines and regulations also introduced an increased amount of work. Cancelled meetings affected the flow of information to the employees and even if the information was sent by email, they did not always have time or energy to open and read it.

Lastly, the use of personal protection equipment also made it more difficult to have contact with the women on the ward, and it became harder to to examine them and provide adequate care. The additional time needed to put the PPE on safely and remove it afterwards was not always there, resulting in stress.

*“It was very cumbersome as well. It takes much longer, just all the protective gear and much more work for the staff.” – Physician in focus group interview 1*

##### Emotional demands

The midwives and physicians described being frustrated when routines and guidelines on how to safely manage care in the wards and how to protect the women and themselves changed from one day to the next. They feared becoming infected by the women or colleagues, especially as some were asymptomatic. The MHC employees also said they worried about bringing the virus home to their own families and to people in risk groups. When the testing of employees, women, and partners was introduced, there were initial doubts about whether or not they were using the correct test material, which was perceived as stressful. However, when the test routine worked and there were clear guidelines about using the protective equipment, this was perceived as positive.

*“[…] the COVID times are just a memory of it being stressful and uncertain.” – Midwife in focus group interview 3*

Working with masks and visors was also found to be hard and frustrating. Both professions described it harder to work in protective equipment because it became too warm, but they were also grateful for the equipment (see below regarding cognitive demands).

Another emotional demand during the pandemic was having to explain to the woman’s partner, the other parent, that they could not accompany her to the post-natal ward, and some partners had a hard time accepting this. When trying to enforce the rules the employees had to face sad and angry parents, and even deal with women who refused to stay in the post-natal ward, although they had a medical indication.

*“This thing about us implementing rules that I didn’t agree with… This thing about dad not being allowed in the post-natal ward. It was very difficult for me because I think it’s an absolutely crazy rule. I didn’t understand, when we see mom and dad as equals… of course, the man should be there in the post-natal ward too.” – Physician in focus group interview 1*

##### Cognitive demands

The requirement to wear protective equipment was experienced as an additional cognitive demand and stood out as the most negative of all requirements, as it formed a barrier between the employees and the women. The midwives said it was hard for the pregnant women to communicate, especially when there were language barriers.

*“The only thing I thought about, in addition to the protective gear, we have quite a bit… and it’s probably generally true in Sweden, there are quite a few people we meet who don’t speak Swedish. Maybe a few words. And then I also think, in that situation, it can be difficult for a Swedish-speaking woman when we’re dressed in our face masks, but I think for those who don’t understand at all, or who aren’t familiar with Swedish healthcare, there are even more communication gaps.” – Midwife in focus group interview 3*

Uncertainty regarding the impact of the virus on society and how it affected the administered care and its quality also increased cognitive demands on the employees, forcing them to cope with feelings of insecurity.

*“… we were so completely unaware of everything, how it was in the whole of society and also with us. And now it’s been a while, but I can remember that there were a lot of question marks and concern among both patients and staff. Then there was a period when we got a bit… we got direction, but it changed quite a few times. And it was difficult. We’re a very large working group that had to keep track of those guidelines, and it was tough. It was very tough. At the same time, there was this uncertainty about how serious it was and how it spread.” – Midwife in focus group interview 2*

Attempts from the management to decrease uncertainty for the employees by increased communication also introduced cognitive demands in terms of having to handle an extensive amount of information.

*“Because we received some every day, I think we got… even though we hadn’t changed any work routines, we always got these statistics every day. So eventually, you got to the point where you were just like, ‘I can’t handle reading another COVID email’ because it was difficult to find… But if it wasn’t something useful for me right then – I didn’t need to know how many were in the ICU for me to get through that evening. That it was a lot, maybe, a bit like… you almost got to the point where you couldn't open those COVID letters anymore, just.” – Midwife in focus group interview 3*

#### Job resources

The subtheme *Job resources* constitutes aspects of the job that were functional in achieving work goals and reducing job demands and their associated physiological and/or psychological costs, or that stimulated personal growth, learning, and development during the pandemic. The subtheme includes four categories: *Working methods and tools, Competence and staffing, Possibility of control, Peer Support.*

##### Working methods and tools

During the COVID-19 pandemic, changes to normal working methods had to be made, and new working methods and tools were introduced to manage the restrictions and maintain normal operations.

Employee meetings or meetings with groups that were responsible for providing pandemic information at the hospitals were changed to digital meetings. To start with, there were concerns about the outcome of the digitalization process. However, later on, there was appreciation for the increased digitalization, as it was possible to attend the meeting from home and avoid big groups meeting in one room. Nevertheless, digital meetings with no direct interaction were perceived as problematic by all the professions, who described a lack of interaction and contact with each other. Digital appointments were also increasingly made with patients, which was perceived as positive.

*“The digital aspect, for me, I find it very convenient.” – Physician in focus group interview 1*

Saving time was a positive aspect linked to certain working methods that had been changed and adopted during the pandemic.

*“Then there are probably some advantages as well, I think. For example, that we can sit like we are doing now, there… A lot of meetings, a lot of digital meetings, and transportation and all the stuff that … So there have definitely been advantages too. And some new routines for us.” – Midwife in focus group interview 2*

In terms of preventative measures, there was a lot of focus on hygiene and concerns about what kind of protective equipment should be used – whether it was wright or wrong. Standard infection control precautions were highlighted, and guidelines from the Public Health Agency of Sweden were implemented at the hospitals, for example, keeping a distance.

*“But it feels quite nice when we have more infection control precautions, that we don’t go around greeting everyone and shaking hands because it’s mandatory to maintaining distance." – Physician in focus group interview 1*

##### Competence and staffing

The recommendation was that employees should stay at home if they were the least bit suspicious they or anyone else in their household had COVID-19 symptoms, which meant that sickness absence increased among staff. As a result, the remaining employees had to work extra shifts, and this, as well as a tangible concern about being asked or ordered to work overtime affected them negatively.

*“Yes, and it was noticeable because… well, sick leave increased because everyone… we were working tirelessly all the time. We were exhausted, and there was a lot of short-term sick leave, really.” – Midwife in focus group interview 3*

##### Possibility of control

Receiving continuously updated information gave employees a feeling of control when navigating uncertain situations. Naturally, they knew how to do their routine work, but had to deal with new situations, if the patients or their colleagues became infected. For example, the guidelines for testing changed several times but the fact that the staff were frequently provided with updates maintained their feeling of control.

*“And that’s why we needed updates. Every week, they had a meeting, all the physicians received information, and then we disseminated this information to our other colleagues. So, it was always ‘what is the current situation now?” – Physician in focus group interview 1*

The employees got new information on a regular basis and appreciated all of it, even if it was too much to take in, especially at the beginning of the pandemic. In order to reach as many employees as possible as quickly as possible, opportunities to inform them about any changed or new guidelines were found in the overlap time between shifts, in weekly letters, and/or the intranet of the organization.

*“I know that in the beginning, I still thought that they were relatively clear with… and came up with new guidelines and that they were available, and that they informed us in many overlaps and so on. So, I still thought management handled it relatively smoothly.” – Midwife in focus group interview 2*

It was clear that the managers in each of the different workplaces/wards tried to reach out to the staff with new information about the pandemic and give them updated guidelines or new ones.

*“They really did the best they could. And even though we could read on the intranet, they were still at every… well, not every, but on many overlapping occasions, during reports and such, informing us about the situation right now because it changed so much. It could be about who was allowed to come or not, and which test tubes to use, and when to wear face masks. There were so many things all the time. I think they did the best they could there. They were very present.” – Midwife in focus group interview 2*

In Sweden, as in other parts of the world, many people became unemployed due to the COVID-19 pandemic. Having a workplace that depended on the employees’ knowledge and skills increased the feeling of control in the uncertain times.

*“People haven’t had to be afraid of losing their jobs, which was talked about in the beginning. It feels like they have completely… in some way, let it go a bit in Sweden because they have still allowed society to keep going. So even though there is high unemployment in some groups and so on, at least we haven’t been worried about not being able to work.” – Midwife in focus group interview 2*

##### Peer support

The pandemic shed light on some important aspects of the workday that the participants had previously taken for granted. For example, the switch to digital meetings meant less contact and spontaneous discussion between the employees.

*“… we used to have reflections at the end of the workday, before going home and all, we used to gather and reflect on how the shift had been, how we were feeling, what we had done, and what we could have done better, and so on. All that stopped. Partly because we weren’t allowed to meet, that is, be too close to each other. Also, because we didn’t really have time for it. There was a very high workload, and all these meetings we had were regularly cancelled. Our training sessions were cancelled, and… Then it got a bit more that it was… These online meetings came later on, but initially, it was… And it got to be a bit word of mouth, but there was never a gathering where we could talk about how we really felt.” –Midwife in focus group interview 2*

The employees found strength in their sense of loyalty to one another, even when they knew they might not be able to finish their shifts on time because many of their colleagues were on sick leave. Despite heavy workload, overtime and uncertainties regarding the virus, there was a sense of security and appreciation for having a job to go to and doing it well.

*“It’s such a loyal workgroup that is somehow a bit used to the fact that you have to support each other and the expectant mothers. Because otherwise, we have to admit, maternity care wouldn’t have functioned at any point. So, this is not only related to COVID, but it became very clear during this period.” – Midwife in focus group interview 2*

The work gave the employees a great sense of meaningfulness, and all the professions were proud of doing an excellent job.

*“All the time, because I think like this: I would have died if I had to sit at home and work for a year and a half. Because I really appreciate having a workplace and getting to be social with other people and stuff. So, I think that we have been allowed to… even if we had to learn not to sit too close to each other when we had lunch, we’ve carried on sitting in the same room with a few people to have lunch. And I am very grateful for that, actually, for my own well-being. I think I would have found it tough to have to sit alone. I’m very happy to have a job where I get to meet people, both patients and colleagues.” –Midwife in focus group interview 2*

### Self-assessed workload and burnout during and after the pandemic

**Fig. 1:**
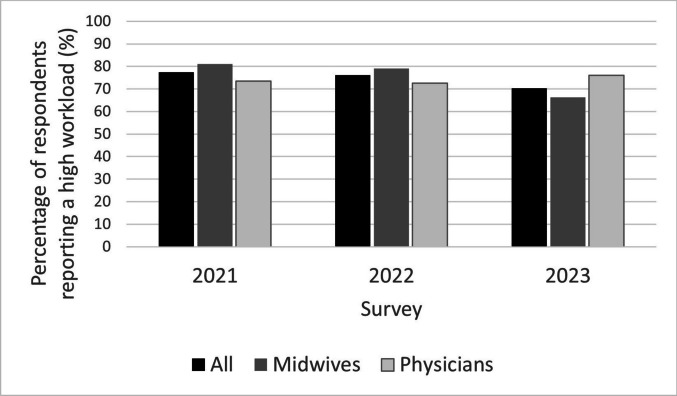
Percentage of respondents reporting a high or a very high workload during (2021 and 2022) and after the pandemic (2023). In total, 782, 503 and 759 maternal healthcare employees responded to the surveys in 2021, 2022 and 2023, respectively. Out of these, 402 (51%), 274 (54%) and 455 (60%) were midwives and the remaining physicians

In the quantitative surveys, 77% (*n* = 782) and 76% (*n* = 503) of the respondents stated they had a high or very high workload during the two measurement periods during the COVID-19 pandemic compared to 70% (*n* = 759) in the survey after the pandemic. Midwives reported slightly higher levels of workload than physicians during the third wave of the pandemic in 2021 (*p* = 0.01, *n* = 401). In 2023, after the pandemic, the opposite was found, with somewhat more physicians reporting a high or very high workload (*p* = 0.004, *n* = 305) (Fig. 1).

**Fig. 2:**
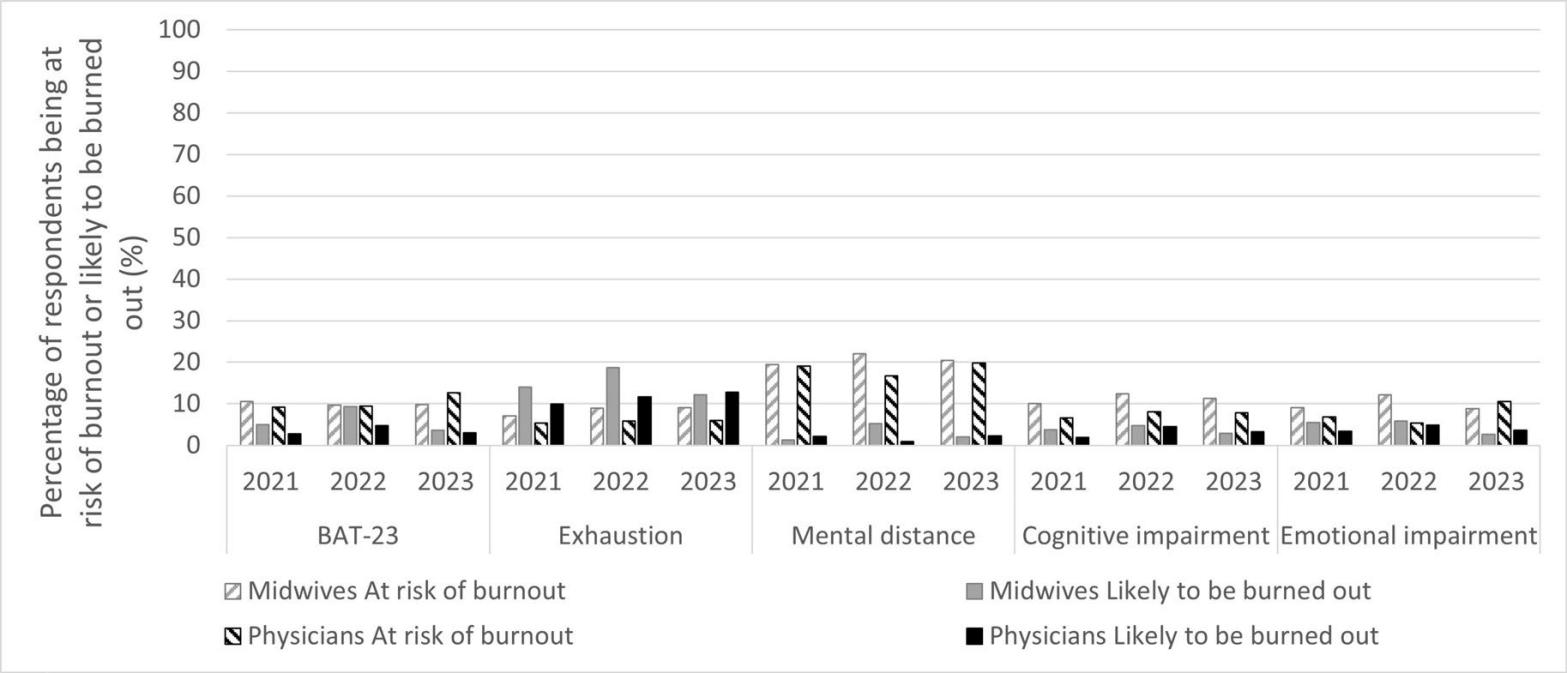
Percentage of respondents at risk of burnout or likely to be burned out during (2021 and 2022) and after the pandemic (2023). In total, 782, 503 and 759 maternal healthcare employees responded to the surveys in 2021, 2022 and 2023, respectively. Out of these, 402 (51%), 274 (54%) and 455 (60%) were midwives and the remaining physicians

The average levels of burnout for BAT-23 were 1.95 (*n* = 748) in 2021, 2.05 (*n* = 470) in 2022, and 2.00 (*n* = 747) in 2023, with 3.9%, 7.2%, and 3.4% found to be likely burned out respectively. In addition, 9.9%, 9.6%, and 11.0% were at risk of burnout in 2021, 2022, and 2023 respectively (Fig. 2). The 2022 survey revealed slightly higher levels of respondents likely to be burned out compared to in 2021 and 2023 (Fig. 2). In 2022, midwives had a slightly higher percentage of being at risk of burnout and/or likely to be burned out than physicians on the subscales exhaustion (*p* = 0.03, *n* = 268), mental distance (*p* = 0.006, *n* = 268) and emotional impairment (*p* = 0.02, *n* = 271) (Fig. 2).

## Discussion

During the pandemic, the Swedish MHC employees experienced many changes to their working routines but managed to maintain operations overall nonetheless. These changes mainly concerned introducing triaging and screening procedures to identify infected cases, restricting the possibility for partners or next of kin to stay on the wards, implementing routines for use of personal protection equipment, and changing to digital meetings with the patients where possible. Similar changes of work routines have been described in Swedish [13] and international studies [10, 14, 16–18] prior to this study.

As a result of these changes, midwives and physicians within MHC experienced increased job demands, comprising a higher number of work tasks and higher emotional and cognitive demands. The main reasons perceived to be behind these increased demands were associated with the implementation of national guidelines, such as limiting the number of individuals in confined spaces, and introducing triaging and screening procedures to identify infected cases. These restrictions aimed to reduce the spread of the disease in society and were mostly experienced as adequate and positive by the employees. However, although perceived as adequate, restrictions on partners within MHC sometimes provoked moral distress among the MHC employees, as they felt unable to act in accordance with their moral values, something that has been examined in previous research [30]. The employee’s situation was further worsened by concern for their own health and that of their next of kin, along with general uncertainty in society during the pandemic, also in line with previous research [31]. During a crisis, maintaining operations within a healthcare sector that cannot postpone or delay care will lead to increased demands on employees. Our findings suggest that the employees perceived their managers had limited scope to reduce these demands, highlighting the need to increase resources during a crisis in order to maintain operations and protect employees. This includes helping them manage their anxiety about the impact of the crisis on their family and society in general. In addition, staff need support in handling situations that might result in moral distress.

To balance these increased demands in the Swedish MHC, new working routines were introduced. Regulatory strategies (i.e. when resources from one domain of life are drawn on to fulfil demands in another domain) were also identified on an organizational, leadership, familial, and individual level [25]. At the organizational level, management made extensive efforts to provide all employees with information about the general COVID-19 situation in their area. This information was mostly communicated via email. Digital solutions for staff meetings and patient appointments, as well as personal protection equipment were provided by the organization. The staff were grateful for these efforts, but sometimes the information burden was too great, making it hard to take in and potentially leading to misunderstanding and confusion.

During a crisis, first-line healthcare managers have an important role in mediating the sensemaking between top managers and employees [32–34]. In our study, the first-line managers had to handle staff concerns, communicate and implement the updated routines, and try to secure adequate staffing despite changing directives and limited scope to control the situation. They did this through a combination of communicating by email and being present during work shifts. Staff experienced this approach as successful, recognizing that their managers were trying their best to maintain normal care for all patients while also caring for them as employees, which was regarded as supportive in the situation. Thus, the organization and healthcare managers had to provide resources, partly to maintain operations and partly to buffer the impact of demands and anxieties on an individual level [25]. However, success was largely driven by individual leaders’ behaviour – the staff experienced very few organizational-level measures aiming to balance increased demands during the pandemic. This may have led to strained working conditions for the managers. Thus, it is clear that during a crisis, first-line healthcare managers need sufficient organizational resources to allow them to support their staff and healthcare organizations [35]. The MHC organizations also need to build organizational resilience that enables them to adapt, learn and develop successfully during times of unpredictability, insecurity, and rapid changes [35]. To do so, experiences from past crises need to be systematically collected and preventive measures need to be identified and implemented.

At the household level, heightened concerns about the health and safety of family members and having to implement stay-at-home guidelines for those with mild symptoms or having to care for children exhibiting such symptoms, required allocation of resources stemming from the job domain. However, resources were also transferred from the family domain to the job domain by enabling MHC employees without symptoms to work longer and extra work shifts. This was also seen in another study, partly using the same study population, where increased work-life conflicts were seen during the pandemic among MHC employees with a high workload [22].

On an individual level, MHC workers tried to balance the increasing demands during the pandemic by increasing their peer support and creating a sense of coherence within the work units. However, despite efforts on multiple levels to increase resources to balance these increasing demands, the employees experienced an increased workload. This was confirmed in the survey where a high percentage of the respondents reported a high workload both during and after the COVID-19 pandemic. Worryingly, the MHC employees also reported high levels of workload after the pandemic, with 70% of the respondents reporting a high workload. In addition, many midwives and physicians reported negative health effects while working during the pandemic. Similar results have been seen internationally [9, 14, 15, 19–21] and in a prior study with partly the same study population, where decreased job satisfaction and increased levels of stress and exhaustion were seen for employees with increased workload during the pandemic [22].

This imbalance between the MHC employees’ job demands and job resources could potentially also lead to more adverse health effects, especially when present over a long period of time [25]. However, our results indicate that the identified regulatory strategies played an important role in mitigating the impact of the pandemic on the operations and staff. The level of burnout found in this study corresponds to levels found for Swedish physicians and nurses surveyed in February to March 2022 during the COVID-19 pandemic using the BAT-12 questionnaire [36] and Swedish midwives surveyed before the pandemic using the BAT-23 questionnaire [37], indicating the pandemic had limited effect on levels of burnout. However, another 10% were at risk of burn out, which indicates further action is required to decrease the workload within MHC. Thus, managers and healthcare organizations need to work systematically to decrease demands on MHC workers while increasing their resources, both during normal operations and crises, to prevent elevated levels of burnout due to adverse working conditions.

A strength of this study is the mixed methods approach with a convergent design [26], where the qualitative analysis enabled us not just to identify changes in the MHC workers job demands and resources but also to identify changes in job demands and resources which the study population experienced as important. The convergent design of the mixed methods approach [26] also gave an opportunity to verify the findings within a larger study population (which the respondents belonged to) on multiple occasions during and after the pandemic, resulting in a deeper understanding of the working conditions for Swedish midwives and physicians within the MHC. Trustworthiness was considered during the preparation phase, organization phase and reporting phase of the qualitative analysis, in accordance with the literature regarding good practice [38]. Regarding limitations, although measures were taken to ensure a variety of participants in the focus group discussions, having a total of 13 respondents from three focus group interviews somewhat limits transferability and it had been beneficial with one more focus group discussion with physicians. In regard to the quantitative analysis, one limitation is the relatively low number of participants, which may affect generalizability of the findings. In addition, the absence of pre-pandemic data affected the possibility to separate the effect of the pandemic from other sources of workload within these professions. However, the repeated measurements and nationwide recruitment increase the credibility of the data.

## Conclusions

The COVID-19 pandemic had a large impact on maternal healthcare staff in the form of changed working routines and increased job demands (mainly a higher amount of work and higher emotional and cognitive demands) resulting in increased workload. Nevertheless, the respondents experienced that MHC succeeded in maintaining operations overall. To balance these increased demands, healthcare organizations added resources, such as new working routines, digital work tools, information and support, but the employees nevertheless experienced an imbalanced workload, with job demands exceeding available job resources. To cope with the situation, regulatory strategies on an organizational, managerial, familial, and individual level played an important role in mitigating the impact on operations and staff. However, these regulatory strategies were mainly driven by the efforts of individual leaders and team members rather than support on an organizational level. To strengthen the resilience of MHC organizations in future crises, organizations need to work systematically to improve midwives’ and physicians’ working conditions overall, and lessons learned from the COVID-19 pandemic need to be used to design support measures on an organizational level that may be put into use in case of a future crisis.

## Supplementary Information

Supplementary Material 1.

## Data Availability

The datasets generated during and/or analyzed during the current study are available from the corresponding author on reasonable request.

## Abbreviations

MHC: Maternal healthcare
JD-R: Job Demands-Resources
