# Supplementary material for: Maintaining operability at a high personal cost – a mixed method study on maternal healthcare workers’ experiences during the COVID-19 pandemic
Source: BMC Health Serv Res. 2025 Jan 29;25:173. doi: 10.1186/s12913-025-12337-0 (PMC11776309; doi:10.1186/s12913-025-12337-0)
Supplement: Supplementary file 1 — Supplementary Material 1. [file 12913_2025_12337_MOESM1_ESM.docx]

Interview guide

The discussion was started by the open question ‘How has the COVID-19 pandemic affected your work situation?’ with follow up questions according to what came up during the discussions.
